# Psychological pain and suicidal behavior: A review

**DOI:** 10.3389/fpsyt.2022.981353

**Published:** 2022-08-22

**Authors:** Ilya Baryshnikov, Erkki Isometsä

**Affiliations:** ^1^Department of Psychiatry, Helsinki University Hospital, Helsinki, Finland; ^2^Department of Psychiatry, University of Helsinki, Helsinki, Finland

**Keywords:** psychological pain, suicide, suicidal ideation, suicidal behavior, psychological theories, psychache

## Abstract

Despite accumulation of clinical research on risk factors for suicidal process, understanding of the mechanisms and pathways underlying the emergence of suicidal thoughts and their progression to acts is insufficient. The suicidal process has been conceptualized in multiple psychological theories that have aimed to shed light on the interplay of contributing factors. One of the central concepts included in both the cubic model of suicide and the three-step theory of suicide is psychological pain (mental pain or psychache). Over the two last decades, interest in psychological pain has increased considerably, particularly since the discovery of the complex link between the pain processing system and the neurobiology of suicide, and the putative antisuicidal effect of buprenorphine. Growing evidence supports the association between experiencing psychological pain and suicidal ideation and acts in both clinical and non-clinical samples. However, many questions related to the concept of psychological pain and its role in prediction of suicidal behavior remain to be answered in future research. In this narrative review, we have outlined the history of the concept, the definition of psychological pain, and the tools developed for its measurement, summarized the empirical research on psychological pain in relation to suicidal behavior, and suggested future directions for clinical research on psychological pain and suicidal behavior.

## Introduction

Suicide is one of the most important contributors to mortality worldwide, representing one of 10 top leading causes of death in Europe and North America (1) and the central cause for deaths among young people. Understanding the suicidal process and recognizing modifiable risk factors for suicidal behavior are vital for suicide prevention.

Over the past decades of research on suicidal behavior, a wide range of risk factors for suicidal ideation and acts has been identified in the general population (2) and in psychiatric settings (3, 4). Although risk factors for suicidal ideation may partly differ from those of suicidal acts, having a mental disorder appears to be one of the most consistent factors for both phases of the suicidal process. In European and North American psychological autopsy studies, about 90% of persons ending their life had suffered from a mental disorder and about 60% from mood disorders (5, 6). The presence of a mental disorder is an important but on its own an insufficient precursor for suicidal thoughts and acts, motivating researchers to investigate other underlying mechanisms of the suicidal process.

As suicide is defined as the act of intentional ending of one’s life, scrutiny of the intrapsychic events prompting and maintaining the process of emergence of suicidal ideation and progression to action is crucial in suicide research. Numerous psychological theories have been created to explain the suicidal process (for review, see O’Connor et al. (7). These theories provide an important framework to conceptualize the suicidal process and identify potential targets for treatment. Among contemporary psychological theories of suicidal behavior, the most important and empirically tested are Beck’s hopelessness theory (8), the Three-Step Theory (9), the interpersonal theory of suicide (10, 11), the motivational-volitional model of suicidal behavior (12) and the fluid vulnerability theory (13). Although partly different, all of these theories are broadly consistent with a stress-diathesis model of suicide, emphasizing a complex interaction between trait-like vulnerability factors and state-dependent stress factors (14). However, despite substantial advancements made in understanding the suicidal mind, prediction of suicidal acts at the individual level is impossible, and only a few evidence-based treatments for suicidal behavior are available. Therefore, there is a need for a more profound understanding of the psychological factors leading to suicide and new treatment targets.

Psychological pain (mental pain, psychic pain, emotional pain, or *psychache*) is one of the concepts included in the current psychological theories of suicidal behavior (such as the Three-Step Theory of suicide, 3ST). This term has been known in suicidology literature for decades since Edwin Shneidman’s formulation of the Cubic model of suicide (15), described also in this paper. Shneidman postulated psychache to be the core of the suicidal process, explaining suicidal ideation, attempts, and completed suicide. Psychological pain is later defined as “an extreme and aversive emotionally based feeling, experienced as a lasting, unsustainable condition resulting from a negative appraisal or deficiency of self” (16). According to Shneidman, as psychological pain becomes unbearable, suicide appears to be the only means to stop the painful internal state for some people.

During recent decades insight into the complex associations between processing of pain, emotions, and suicidality has evolved. Previous studies have shown a significant overlap in neural systems mediating emotional and physical components of pain (17, 18). The endogenous opioid system is demonstrated to largely participate in processing of human emotions (19) and neurobiology of depression (20). Moreover, the experiences of social rejection or separation, common life events preceding both onset of depression (21) and suicidal behavior (22), seem to activate the brain network influencing the affective and sensory component of pain processing (23). Reduced tonic opioid activity is suggested to be associated with dysphoria and suicidal behavior in patients with borderline personality disorder (24). Finally, a promising randomized controlled trial on potential effect of low-dose buprenorphine on suicidal ideation in patients with depression (25) has reawakened interest in the relation between psychological pain and suicidality, leading to growing empirical research on psychological pain in the suicidal population (26).

The only meta-analysis of studies on the relationship between psychological pain and suicidality in both the general population and clinical samples revealed higher psychological pain in subjects with current and lifetime suicide attempts and suicidal ideation (27). However, many questions remain to be elucidated in future research. Among these questions, the predictive value of psychological pain in relation to suicidal ideation requires more prospective studies, and the associations between psychological pain and such well-known risk factors for suicide as a severity of psychiatric pathology or hopelessness in relation to suicidal ideation and acts need to be clarified.

The aims of this paper were a) to review the literature on the history of the concept “psychological pain” and its definition and measurement; b) to briefly review the empirical research on the association between suicidal behavior and psychological pain in both general population and clinical samples; and c) to outline key directions for future psychological research.

## Concept of psychological pain

The concept of intrapsychic pain and its association with suicidality has fascinated writers, philosophers, and poets through the centuries. Research on psychological pain as a risk factor for suicide in psychology and psychiatry had long been scarce, accumulating only within the last few decades. The most extensive contribution to the conceptualization of psychological pain in relation to suicide has been provided in Shneidman’s Cubic model of suicide.

### Shneidman’s “cubic model of suicide”

Edwin Shneidman, an American clinical psychologist and suicidologist, introduced the term “psychache” and postulated that psychache (psychological pain) is a central precondition for suicide as a result of his intensive 40-year autopsy studies of suicide completers (15). Shneidman stated that the most common sentence in suicide notes was “I can’t stand the pain any longer”, which led him to conclude that suicides are caused by psychache, referring “to the hurt, anguish, soreness, aching, psychological pain in the psyche, the mind” (28), or “the pain of excessively felt shame, or guilt, or humiliation, or loneliness, or fear, or angst, or dread of growing old, or of dying badly, or whatever”. “Hopelessness” and “helplessness” are common “emotions” involving in suicidal crisis. According to his work, suicide is a conscious practical act intended to stop the unbearable flow of intolerable pain, indicating different individual thresholds for enduring psychological pain. Shneidman hypothesized that psychache is created and sustained by thwarted, unfulfilled, or frustrated psychological needs, referring to a theory of personality (personology) developed by Henry Murray and positing an individual hierarchy of psychological needs driving one’s behavior (29). Among these needs, central in terms of suicide are the need for dominance (to control, influence, and direct others), autonomy (to be independent and free, shake off restraint, to resist coercion and restriction), counteraction (to master a failure or loss by restriving, to obliterate a humiliation by resumed action), succorance (to have one’s needs gratified by the sympathetic aid of another person, to be taken care of and loved), affiliation (to please and win the affection of a respected person), and order (to put things and ideas to order, to achieve arrangements, organization, balance, tidiness, a precision in the outer world or ideas in the inner world).

Shneidman illustrated his model of suicide as a cube with three visible facets, representing three components of this model: “pain,” “perturbation,” and “press,” the severity of each varying from “1” (“little pain,” “low perturbation,” and “positive press”) to “5” (intolerable pain,” “high perturbation,” and “negative press”). “Perturbation of the mind” is the state, occurring when an individual experiences an intolerable psychache, and refers to being upset, agitated or perturbed, potentially also indicating the presence of a mental disorder. “Perturbation” includes transient perceptual constriction (a cognitive distortion related to seeing suicide as the only solution for the unbearable pain) and “a penchant for precipitous self-harm or inimical action” (a characteristic akin to impulsivity). “Press” includes both actual and imagined stressors. In this model, only persons scoring 5 in terms of all three components kill him- or herself and alleviating severity of even one of these may reduce the suicide risk.

### Development of the concept

The concept of psychological pain has evolved over time. Roy F. Baumeister in his theory “suicide as escape from self” (30), although not directly using the term “psychological pain”, referred to a painful awareness of self’s inadequacy, which in turn generates negative emotions and a strong desire to escape this state, bringing irrationality and inhibition, “making drastic measures more acceptable” (30). Orbach et al. (31) referred to an “unpleasant feeling of negative changes in the self” and in their studies on student samples using the factor analysis of the 44-item Orbach and Mikulincer Mental Pain Scale (OMMP) distinguished nine dimensions of psychological pain: loss of control, narcissistic wounds, freezing, irreversibility of pain, emotional flooding, estrangement, confusion, social distancing, and emptiness (31). Eisenbeger (32) described “social pain” – an intrapsychic unpleasant experience related to actual or potential social rejection or exclusion – and demonstrated activation of a neutral network involved in the processing of psychic pain. Finally, Meerwiijk (16) suggested a consensual definition of psychological pain as follows: “a lasting, unpleasant, and unsustainable feeling characterized by a perception of inability or deficiency of self”.

### Psychological pain in the Three-Step Theory of suicide

The concept of psychological pain is also central in the Three-Step Theory (3ST) of suicide (33), postulating that the combination of psychological pain and hopelessness causes suicidal ideation. In other words, in contrast to Shneidman’s theory, psychological pain alone is not a sufficient precondition for emergence of suicidal ideation. When pain overwhelms connectedness, suicidal ideation intensifies and progresses to suicide attempts, if the capability of suicide is present. However, as opposed to other theories, the 3ST does not provide a strict definition of psychological pain and the authors emphasize a partial conceptual overlap of psychological pain with other factors often involved in suicidal crisis such as “most mental disorders,” general distress, financial distress, burdensomeness, etc. Moreover, the concept of psychological pain in the 3ST may also include physical pain (33). Similar to the Cubic model of suicide, the 3ST emphasizes that psychological pain needs to be perceived as unbearable to interplay with hopelessness and result in suicidal ideation.

The definition of psychological pain thus remains elusive and seems to overlap with other important concepts in suicide prediction such as “depression,” “hopelessness,” “anxiety,” “general distress,” and even physical pain. All of these factors make measurement of psychological pain and generalizability of study findings challenging.

## Measurement of psychological pain

### General aspects of measurement in psychiatry

The ultimate goal of measurement in psychology and psychiatry is to operationalize theoretical concepts into measurable observations that can be reliably utilized in clinical practice and research. One of the two main characteristics of a measurement tool is its validity, that is, whether the tool measures what it is intended to measure. To define the validity of a measurement tool, several methods can be used, including criterion (i.e., the correspondence of a scale to a true state or gold standard), construct (i.e., the consistency between scales having the same theoretical definition in the absence of a gold standard), and content validity (i.e., the completeness of a scale in coverage of a concept) (34). The other major characteristic of a measurement tool is its reliability, that is, whether the assessment tool consistently measures the phenomenon it is intended to measure. The methods commonly used to estimate reliability of a test include test-retest correlation, internal consistency, and inter-rater reliability.

When measuring a relation between a risk factor and an outcome, it is important to account for a) the temporal stability of an outcome and b) state and trait characteristics of a risk factor (35). As suicidal ideation is known to fluctuate over time (36), the predictive value of its proximal risk factors appears also temporally unstable and state-dependent (36–38) making repeated measurements essential. A risk factor may represent, however, also a temporally more stable trait-like characteristic, indicating a tendency to experience psychic phenomena over lifetime, helping to identify persons “at risk”. Moreover, both the Cubic model of suicide and 3ST, wherein psychological pain plays a key role, highlight the importance of a person’s ability to tolerate psychological pain, as psychological pain needs to be “unbearable” to evoke the suicidal process. Therefore, in this section, we aimed to describe the most frequently used tools for measurement of psychological pain and to define how a tool assesses a) current psychological pain, b) lifetime tendency to experience psychological pain, and c) tolerability of psychological pain (see Table 1).

**TABLE 1:** Assessment tools developed to measure psychological pain.

Name	Description	Assessment of psychological pain			References
		Current	Lifetime tendency	Tolerability	
Psychological Pain Assessment Scale (PPAS)	Includes a definition of psychache and the Thematic Apperception Test (responders are presented with 10 pictures and asked to rate psychache experienced by the main heroes); assesses intensity of current and lifetime worst psychache on a scale from 1 to 9, and feelings prominent in the worst pain; and requests the method used in a suicide attempt and a rating of lethality from “very low” to “close to dying”	Yes	Yes	No	(39)
Orbach and Mikulincer Mental Pain (OMMP)	A self-rating 5-point Likert-scale with 44 items, including nine factors: a) irreversibility, b) loss of control, c) narcissist wound, d) emotional flooding, e) freezing, f) self-estrangement, g) confusion, h) social distancing, i) emptiness; conceptualizes psychological pain as a perception of negative feeling	Yes	No	Yes	(31)
Psychache Scale (PAS)	A 13-item scale, with each item coded with a 5-point Likert score	No	Yes	Yes	(44)
Visual Analog Scale for Psychache	A visual analog scale from 0 to 10 on psychological pain currently and during the last 15 days	Yes	No	No	(54)
Mee-Bunney Psychological Pain Assessment Scale (MBPPAS)	A 10-item self-report scale with 5-point Likert items, assessing both frequency and intensity of psychological pain	Yes	Yes	Yes	(58)
Psychic Pain Scale (PPS)	A 20-item scale based on Maltsberger’s theory of suicidality. The PPS assesses frequency of a range of negative affects on a 5-point Likert scale; two factors: affective deluge and loss of control	N.d.^1^	N.d.	N.d.	(80)
Unbearable psychache Scale (UP3)	A 3-item scale, derived from the PAS, for rating the unbearableness of psychological pain	Not directly	No	Yes	(62)
Mental Pain Questionnaire (MPQ)	Assesses psychological pain “in the past weeks” with 10 items and yes/no responses	Yes	No	No	(81)

1 – Not defined.

### Psychological Pain Assessment Scale

The Psychological Pain Assessment Scale (PPAS) is the first tool created by Shneidman to evaluate psychological pain in suicidal subjects (39). The PPAS includes (a) a brief definition of psychological pain (“mental suffering, inner torment (…) the pain of shame, or guilt, or grief, or humiliation, or hopelessness, or loneliness, or sadness, or anguish (…). It is an ache in the mind”); (b) an item on the intensity of current psychological pain using the Likert Scale 1-9; (c) the Thematic Apperception Test (TAT) (requirement to rate psychological pain that the responder sees in the main characters of five pictures); (d) an item on the worst lifetime psychological pain (Likert Scale 1-9); (e) a requirement to select which of 28 feelings are associated with the worst pain; (f) an item on previous suicide attempts and lethality of the method(s) used; and (g) a requirement to write an essay on the worst psychological pain). The PPAS requires much time to complete and a trained operator to provide and interpret the test. The PPAS does not assess tolerability of psychological pain and includes non-countable items. Moreover, the TAT as well as the essay requirement may be too demanding for patients with severe psychiatric pathology. Several studies utilized the PPAS in clinical samples (40, 41, 42) and one study in a student sample (43). Leenaars et al. (43)and Pompili (42) have discussed construct and content validity issues of the PPAS.

### Psychache Scale

The Psychache Scale (PAS), developed by Holden et al., is a 13-item scale also based on Shneidman’s theory (44). The first nine items cover the presence and frequency of psychological pain, coded on a 5-point Likert scale (from “never” to “always”), and the rest of the items explore how a responder can tolerate the pain (from “strongly disagree” to “strongly agree”). Several studies have shown an excellent internal consistency of the PAS in student populations (44–46) homeless men (47), and small samples of psychiatric patients (48, 49, 50). However, as the PAS does not specify the time frame, it may be difficult to assess the intensity of current psychological pain within a short time, and therefore, may be challenging for repeated measurement.

### Orbach and Mikulincer Mental Pain Scale

The Orbach and Mikulincer Mental Pain Scale (OMMP) conceptualizes psychological pain as a negative change in the self and its functions and includes 44 self-report items on current psychological pain on a 5-point Likert scale (1 = *describes me not at all* to 5 = *describes me very well*). The items are heterogeneous and load on nine factors: (a) irreversibility, (b) loss of control, (c) narcissist wound, (d) emotional flooding, (e) freezing, (f) self-estrangement, (g) confusion, (h) social distancing, and (i) emptiness (31). The OMMP has been used in a series of studies on inpatients (see, for example, Gvion et al. (51); Levi et al. (52); van Heeringen et al. (53), showing good internal consistency. However, the OMMP does not assess lifetime tendency to experience psychological pain and includes a relatively large number of items, loading on a complex set of factors, making it difficult for clinical use. In addition, several items of the OMMP seem to overlap with such known risk factors of suicidal behavior as hopelessness (“I have no idea what to expect in the future,” “I have no future goals,” “I can’t change what’s happening to me”), borderline personality disorder features (“There are strong ups and downs in my feelings,” “I am flooded by many feelings”) or cognitive constructs related to depression.

### Psychological and Physical Pain Visual Analog Scale

Olié et al. (54) developed the Psychological and Physical Pain Visual Analog Scale (PPP-VAS) (54)for measurement of current, mean, and worst psychological pain during the last 15 days, suicidal ideation, and current, mean, and worst physical pain during the last 15 days on a scale of 0 to 10. The PPP-VAS was shown to have a good discriminative validity when distinguishing healthy controls from depressed patients (55). The PPP-VAS was also utilized in two recent prospective studies on psychological pain and suicidality (56, 57). Although the PPP-VAS does not define psychological pain or assess a person’s ability to tolerate psychological pain, it enables differentiation of psychological pain from physical pain and seems to be suitable for everyday use.

### Mee-Bunney Psychological Pain Assessment Scale

The Mee-Bunney Psychological Pain Assessment Scale (MBPPAS) is a ten-item self-report scale that assesses both the frequency and intensity of psychological pain during the last three months and at the moment on a 5-point Likert scale. It also includes an item on how much more psychological pain a person is able to tolerate before it becomes unbearable, two items comparing psychological and physical pain, and an item on thoughts of death as the only way to stop the psychological pain. The authors have tested the MBPPAS in a sample of patients with depression (58) or substance use disorder (59) and in a sample of suicidal veterans (60). In a Turkish study on patients with mood disorders, the MBPPAS showed a good internal consistency (61).

### Unbearable Psychache Scale

The Unbearable Psychache Scale (UP3) includes three statements on tolerability of psychological pain derived from Holden’s Psychache Scale. The UP3 was also shown to have excellent internal reliability and strong convergent validity by the authors (62). However, the UP3 does not directly assess the intensity of current or previous psychological pain.

As many authors have discussed, the validation process of the measurement tools for psychological pain is challenging due to lack of a gold standard. Some authors have used discriminative validity, differentiating healthy controls and patients (55) (61), ideators and non-ideators, and suicide attempter and non-attempters (44, 55), convergent validity (with measures of depression or anxiety) (44, 55, 58, 61), and, more rarely, concurrent validity methods (61). For example, Demirkol et al. (61)found the correlation between MBPPAS and PAS in patients with depression to be only 0.484. Numerous measurement tools theorize psychological pain in their own way, but data on the congruency of these tools is sparse. In other words, it is not evident whether these assessment tools measure the same psychic phenomena.

Overall, although multiple measurement tools for psychological pain are currently available, some of these (PPAS, OMMP) are time-consuming and require an experienced operator to administer and interpret the tests. Some of the tools assess intensity of current (e.g., PPAS, OMMP, PPP-VAS, and MBPPAS) and lifetime psychological pain (e.g., PPAS and MBPPAS), while others assess tolerability of psychological pain (PAS, MBPPAS, UP3), a component that is considered crucial in Shneidman’s theory and 3ST.

## Research on psychological pain predicting suicidal ideation and acts

Although efforts have been made to elucidate mechanisms of psychological pain (53, 63), no consistent biomarkers for psychological pain exist. Therefore, understanding of associations between psychological pain and suicidal behavior relies on clinical observation.

Research on psychological pain and suicidality has grown notably since 1994 (26). The meta-analysis of Ducasse et al. (27) reviewed 20 observational cross-sectional case-control studies examining the difference in psychological pain between individuals with and without current or lifetime history of suicidal ideation or suicide attempt, revealing higher psychological pain in both subjects with current and lifetime suicidal ideation and suicide attempts (27). As the authors noted that the association between psychological pain and suicidality remained significant even when depression levels were not different between subjects.

When examining risk factors for suicidal ideation, suicide attempts, or completed suicide, the prospective nature of the study, among other factors, is important. Prospective studies allow not only estimation of the predictive value of a risk factor in relation to an outcome but also examination ofe its associations with other known risk factors. While numerous cross-sectional studies on associations between psychological pain and suicidal behavior exist, we identified only several follow-up studies that are briefly outlined below.

### Studies including non-clinical samples

In a two-year prospective study with high-risk undergraduates who completed the PAS, Beck Hopelessness Scale (BHS) (64) and Beck Depression Inventory (BDI) (65) at baseline and two years later, a strong correlation emerged between PAS, BDI, and BHS (66). In a linear regression analysis, predicting suicidal ideation both at baseline and during a two-year follow-up, only psychache remained statistically significant, while depression and hopelessness became non-significant after controlling for psychache. Similar results emerged in another four-year prospective study on psychache and suicide ideation in 82 undergraduates (67). A longitudinal study on 611 undergraduate students identified that changes in both psychache (measured with PAS) and hopelessness predicted changes in suicidal ideation at the four-month follow-up. The authors hypothesized that psychache plays a mediating role in the relation between hopelessness and suicidal ideation (68).

### Studies including clinical samples

Prospective studies on psychological pain and suicidal behavior in clinical samples are briefly overviewed in Table 2.

**TABLE 2:** Prospective studies on psychological pain in psychiatric patients.

Study	Sample characteristics	Assessment of psychological pain	Follow-up time	Key findings
Trakhtenbrot et al. (69)	153 psychiatric inpatients with mood disorders and schizophrenia	OMMP	(mean) 5.6 years	Depression and hopelessness, but not mental pain, predicted a follow-up attempt. Hopelessness predicted also medical severity of follow-up suicide attempt.
Tsai et al. (70)	190 inpatients with mood, psychotic, and personality disorders	PAS	3 months (baseline, 4 weeks, and 3 months)	A moderate or strong correlation existed between hopelessness and psychological pain at all three time points. Both PAS and BHS predicted suicidal desire at 4 weeks and 3 months, even after controlling for depression.
Alacreu-Crespo et al. (57)	372 inpatients with major depressive disorder	PPP-VAS	1 year	The patients with higher worst and mean psychological pain at baseline had higher odds of reporting suicidal events during follow-up. The baseline worst psychological pain predicted suicide events also at 6 months. The current psychological pain level and physical pain were not significant predictors of suicidal events
Alacreu-Crespo et al. (56)	372 inpatients with major depressive disorder	PPP-VAS	1 year	Depression severity and worst physical pain predicted reporting psychological pain. Changes in depression severity and physical pain predicted a change in psychological pain. Such depressive symptoms as guilt, loss of appetite, and lack of initiative explained maximal psychological pain.
Ballard et al. (72)	108 psychiatric patients with major depressive disorder and bipolar disorder from the ketamine clinical trial	A Negative Cognition Score	3 – 11 days	Psychological pain and hopelessness were not associated with suicidal ideation in the 3- or 11-day analyses.

In a prospective cohort study of 153 psychiatric inpatients (mean follow-up 5.6 years), psychological pain, measured by the OMMP, did not predict future suicide attempt, as opposed to levels of depression and hopelessness (69).

A prospective study on validity of the 3ST included 190 psychiatric inpatients (including patients with mood, personality and psychotic disorders) and assessed psychological pain with the UP3 at three time points (baseline and at 4 weeks and 3 months postdischarge). The authors showed a strong correlation between psychological pain and hopelessness, and, even more strongly, low belongingness and burdensomeness. To support the validity of the 3ST, the authors demonstrated that the interaction of the UP3 and BHS explained 68% of the variance in suicidal desire at baseline, even after controlling for depression and checking for multicollinearity. Moreover, psychological pain at baseline predicted suicidal desire at the 4-week (*r* = 0.53) and 3-month (*r* = 0.51) time points. When controlling for baseline depression and lifetime ideation, the partial correlations between baseline psychological pain and suicidal desire slightly decreased: at 4 weeks (rp = 0.29, *p* = 0.002) and 3 months (rp = 0.38, *p* < 0.001). Moreover, no statistical interaction between pain and hopelessness was found in the prediction of future suicidal desire at either the 4-week or 3-month time point (70).

Alacreu-Crespo et al. (57) in their one-year follow-up study on 372 inpatients with mood disorders reported that after adjusting for depressive symptomology (history of previous suicide events, medication, and psychiatric comorbidity) the patients with higher worst psychological pain in the 15 days (assessed with PPP-VAS) at baseline had higher odds for suicidal events (including suicide deaths, suicide attempts, and hospitalizations due to suicidal ideation) during the one-year follow-up (OR = 1.27) (57). The authors also noted that patients with a recent suicide attempt report higher worst psychological pain during last 15 days (assessed with PPP-VAS) than non-attempters and past suicide attempters, even after adjusting for psychic pain and depression. Moreover, depression severity, along with physical pain, but not hopelessness, predicted high psychological pain, and changes in depression severity predicted changes in psychological pain during follow-up. In addition, such depression symptoms as guilt, lack of initiative, and loss of appetite explained maximal psychological pain in patients with mood disorders (56).

Finally, the latest report from a prospective study on 108 psychiatric patients recruited into a ketamine clinical trial showed that psychological pain [measured by a Negative Cognition score, comprising items from different depression scales (71)] and hopelessness were not prospectively associated with re-emerging suicidal ideation after ketamine infusions in short-term (3 days) or long-term (11 days) analyses (72).

Overall, clinical research with a prospective design on the association between psychological pain and suicidal behavior is sparse. The studies are methodologically highly heterogeneous and have yielded controversial results.

## Discussion

In this narrative review, we have discussed the concept of psychological pain and its interpretation by different psychological schools, briefly described the most commonly used methods to measure psychological pain and summarized the current empirical evidence on the association between psychological pain and the suicidal process, with a particular focus on prospective studies. Overall, the increasing volume of research indicates that the concept of psychological pain is undoubtfully interesting and may be relevant in suicidology. However, there are several factors that challenge the current understanding of the associations between psychological pain and suicidal behavior.

### Conceptualization of psychological pain

The concept of psychological pain is complex, and its definition remains elusive. The conceptualization of psychological pain differs among psychological theories, resulting in a high number of different measurement tools, the congruency of which needs to be elucidated. The measurement tools used in the prospective studies on the association between psychological pain and the suicidal process interpret psychological pain within their theoretical frames, not necessarily overlapping in terms of content (e.g., PAS and OMMP). Moreover, while the PAS and OMMP define psychological pain by multiple explicit items, the PPP-VAS does not define psychological pain at all, i.e., patients understand the concept in their own way. In addition, the time frame of the assessment tools is not identical. For example, while the PAS does not specify intensity of the psychological pain “at the moment,” the PPP-VAS and OMMP assess current psychological pain. The UP3 does not assess the intensity of current psychological pain, rather how unbearable the current psychological pain is. Additionally, although two theories, the cubic model of suicide and the 3ST, highlight that psychological pain should be unbearable to cause suicidal ideation (in combination with hopelessness in the 3ST), few measurement tools assess how unbearable the current pain actually is. Thus, as the congruent validity of the measurement tools is uncertain, generalization of the findings from these studies may be questionable.

### Outcome of clinical research

The risk factors for suicidal ideation and suicide attempts overlap but are partly different (4). Therefore, the predictive value of psychological pain in relation to suicidal ideation and suicide attempts should be assessed separately in future studies.

### Timing of measurement of psychological pain

Temporal variability of a risk factor should be considered when assessing its predictive value in terms of suicidal behavior (35). While a trait factor representing vulnerability for suicidality can be used to identify patients at risk regardless of the time of the measurement, a state factor represents a disorder-related characteristic. Thus, timing of measurement may be crucial for obtaining valid estimates of risk for temporally variable, state-related risk factors. For example, in our five-year prospective study of patients with depressive disorders, we showed that hopelessness, an evident risk factor for completed suicide, displays a notable temporal variability, worsening during depression and alleviating (but not always fully disappearing) after recovery (73). As psychological pain seems also to grow when severity of depression increases (56), the predictive value of psychological pain may be different during depression than during recovery. Ultimately, it is important to know whether and how much systematic estimation of psychological pain in currently depressed patients with mood disorders improves the clinician’s ability to predict suicide. We are not aware of any studies examining trait and state characteristics of psychological pain in psychiatric patients. In other words, it remains unknown whether experiencing psychological pain is a short-term predictor of suicidality or whether a lifetime tendency of experiencing psychological pain indicates a vulnerability to suicidality.

Ecological momentary assessment (EMA) studies have demonstrated that many known risk factors seem to predict changes in suicidal ideation over months or years, but do not predict over hours and days (36), although findings are not consistent (38). Whether psychological pain plays a role in short-term prediction of suicidal ideation and suicide events is a topic for future EMA studies. Understanding whether psychological pain represents a state or trait characteristic (or both) may help researchers to delineate a place for psychological pain in suicide prediction.

### Conceptual overlap with other risk factors for suicide

The strong correlations between hopelessness, depression, and psychological pain, reported by numerous studies, are probably underpinned by partial conceptual overlap between these psychic phenomena. Therefore, when assessing predictive value of psychological pain in relation to suicidal behavior, it is important to elucidate the interactions between psychological pain, depression, and hopelessness. We have previously demonstrated that the predictive value of hopelessness in terms of suicidal ideation notably decreased after adjusting for depression severity (37). The current evidence of the relation between severity of depression, hopelessness, and psychological pain is scarce and partly controversial, requiring elucidation in different clinical samples.

### Psychological pain in patients with borderline personality disorder

Psychological pain may present differently in different diagnostic samples. Patients with borderline personality disorder (BPD) are known to display affective instability due to marked reactivity of mood (74) with an intense episodic dysphoria commonly associated with interpersonal disputes (75). A recent cross-sectional observational study on 2,137 psychiatric patients indicated that suicide attempters had a higher risk for reporting worse mental pain (assessed by the PPP-VAS) and having BPD. In this study, mental pain mediated the relation between childhood traumatic experiences, an important etiological factor also in BPD, and suicide attempts (76). Previously we have shown that features of BPD mediate the total effect of childhood maltreatment on lifetime suicide attempts in patients with mood disorders (77). Thus, both mental pain and BPD represent a risk factor for suicide attempts, and both associated with childhood traumatic experiences. Moreover, opioid system dysfunction is suggested to serve a crucial role in alerted affiliative behavior of patients with BPD (24). In other words, these patients may experience more intensive psychological pain associated with interpersonal conflicts or rejection, increasing their vulnerability for suicidal behavior. Evidence supporting this is, however, scanty, and partly inconsistent (78, 79), motivating future investigations.

### Tolerance of psychological pain

Finally, the cubic model of suicide and the 3ST postulate that psychological pain needs to be experienced as unbearable to launch the suicidal process. Therefore, future research should elucidate whether measurement of individual capacity to tolerate psychological pain would be a better predictor of suicidality than measurement of the current intensity of psychological pain. An improvement in capacity to tolerate psychological pain may thus be an important target for suicide prevention.

To conclude, the concept of psychological pain has received much attention over the last decades, likely representing an important modifiable risk factor for suicidal behavior, but its role in prediction of suicidal ideation and suicidal acts must be clarified. Challenges related to research on psychological pain may be the term’s variable definition and differences in its conceptualization by different psychological schools, resulting in numerous measurement tools. Some of these currently available measurement tools include large numbers of items, which may be demanding for clinical use, and only a few assess the tolerability of psychological pain, a crucial factor in theoretical models. There is also a conceptual overlap with well-known robust risk factors for suicidal behavior, such as “depression,” “hopelessness,” “anxiety,” and even physical pain, indicating a significant correlation and eventual covariation of psychological pain with these risk factors. Therefore, the definition of psychological pain, its common conceptualization by patients and clinicians, and distinguishing it from, for example, emotional and cognitive constructs related to depression or anxiety may be difficult and should be elucidated in subsequent studies. Estimating its predictive value for suicidal ideation and acts in different diagnostic groups of psychiatric patients independently of the robust risk factors (severity of depression, hopelessness, anxiety, etc.) should be a core goal for future research.

## Author contributions

IB reviewed the existing literature and wrote the manuscript. EI provided critical comments on the review.
